# Atlanta Society of Medicine

**Published:** 1886-02

**Authors:** 


### ATLANTA SOCIETY OF MEDICINE.

Officially Reported for The Atlanta Medical and Surgical Journal.

Stated Meeting, January 5, 1886.

The meeting was called to order by the President, Dr. J. D. Wilson.

Dr. Wile reported two cases of eczema fissures.

In both cases the hands, and especially the dorsal surfaces, were involved, the process extending on to the fingers in the form of a chronic inflammatory infiltration, accompanied by the development of deep and painful fissures or cracks over the articulations of the phalanges. One of the cases was on a woman about thirty-five years old, having existed six years. The other on a young man twenty-four years of age, existing nine months. In both cases there was considerable thickening of the epidermis, and in the young man the process invaded the palm of the right hand, forming on the terminal side of the palm toward the articulation of the index and middle fingers a sharply circumscribed callous lesion. He spoke of the difficulties which palmar cutaneous affections often present in the matter of differential diagnosis when they existed alone, as the majority of them are of specific character. But here the presence of the painful fissures on the fingers and the squamous lesion on the back of the hands, together with the history and course of development, plainly indicated an eczematous process.

The method of treatment was in the main the same in both cases.

The indications were first to remove the greatly hypertrophied epiderm, and this was effected by means of sapo viridis spread on muslin rags, applied to the part, allowing it to remain overnight. In the morning the parts were washed with hot water and during the day treated with oxide of zinc ointment. This process was repeated several nights, and most of the horny epidermal layers macerated by the sapo viridis came away. The lesion on the palm of the young man was, however, so callous that several applications of liquor potassa were made. When the horny layer of the epidermis was almost completely removed, exposing here and there the delicate rete layers, zinc ointment was used a few days to allay irritation and this was followed by the use of a paste containing salicylic acid, powdered starch, oxide of zinc, each one-half drachm, vaseline six drachms.

This was used night and day, being freshly applied every morning after bathing the lesions in hot water. When the last ointment was ordered, the patients were also directed to provide themselves with rubber gloves and wear them as much as possible. By means of rubber gloves the moisture or perspiration is confined, evaporation is prevented and the action upon the epidermis is favorable. This disease is always worse in cold weather. Both patients presented themselves about the same time during the month of November, and they are at present free from the affection.

He has used this method of treatment before, but never had such happy and prompt results as in these cases.

Dr. Elkin asked if it was not sometimes difficult to distinguish between the papulo-squamous syphiloderm and squamous eczema of the palms of the hands.

Dr. Wile said that in some cases diagnosis might present difficulty, but bearing in mind the distinct and peculiar development of each of these diseases in this locality, there should be no trouble as to the diagnosis. The eczematous disease always begins in the form of visicles and the squamous stage is the secondary manifestation. A few of these vesicles may still be present, especially in the interdigital spaces, whereas the syphilitic process begins in the form of papules, and they remain as such until their absorption, accompanied by induration and slight desquamation. Furthermore, lesions of syphilis have a peculiar crescentic or circinate configuration.

Dr. Todd stated he was impressed by the pertinence of the question by Dr. Elkin, and that he was inclined to believe with the great Hebra that where you find a diseased condition of the palm, be it on the delicate hand of the princess or the callous hand of the peasant, it is syphilitic.

Dr. Gray reported a case of papilloma of the larynx taken from a child three years old. He first saw the case in November, 1884, and referred it to Dr. Calhoun, but the diagnosis was never fully established during life. The child grew worse, and finally presented symptoms of asphyxia, and then died on account of the mechanical obstruction of the tumor. Dr. Gray also presented the specimen showing the papilloma *in situ*.

Dr. Elkin reported a case of cerebral syphilis. The initial lesion in this case occurred on the index finger of the right hand of a physician sixty-three years of age, at a short interval after making a vaginal examination of a woman of doubtful character. The lesion did not excite suspicion till after an erythematous eruption made its appearance. Then he began to take constitutional treatment, but without any regularity or system. Eight months after the initial lesion, nervous symptoms began to appear in the form of tremors of the hands, insomnia, headache, loss of memory (slight), and later on iritis. His condition had become so that he concluded to abandon his practice and undergo a systematic course of treatment. Hoping to get relief from the baths of Hot Springs, he started on a journey for that point and was interrupted on the way, being attacked by a stroke of paralysis, causing complete paresis of the muscles of the left forearm. There was also a tremor of the lips and slight impairment of articulation and deglutition. There was in addition a heavy feeling accompanied by a slight loss of muscular power in the left leg. He gave up the journey and came to Atlanta with his family. Drs. Todd and Wile saw the case in consultation, and it was decided to place the patient upon proper constitutional treatment. This consisted at first of the internal administration of a saturated solution of iodide of potash, beginning with eight drops and increasing rapidly to forty, three times daily; also inunctions at first of mercurial ointment, later of oleate of mercury, twenty per cent. This treatment, in the course of a week, was followed by marked improvement, the patient being able to move again the wrist and fingers of the palsied hand. After this there occurred a sudden change, characterized at first by unusual restlessness, mental worry, insomnia and other symptoms of general nervous excitement. This was followed after twenty-four hours by a corresponding depression, which suddenly passed into a state of profound coma, from which he could with difficulty be aroused.

This condition continued for thirty-six hours, when death ensued. The case was of unusual interest on account of the early manifestation of the nervous symptoms, although cases are on record where these symptoms occurred six months after the initial lesion. They are, however, rare. The case was undoubtedly of specific character, and from the suddenness of the onset of nervous symptoms there was no doubt as to the occurrence of a cerebral hemorrhage, due either to a degeneration of the blood vessels or the erosion of a blood vessel wall from the extension of a syphilitic gummatous deposit.

Dr. Todd stated that he confirmed what was said by Dr. Elkin, and wished to say in addition that there were distinct, well marked mucous patches on the tongue, which, together with other symptoms, left no doubt as to the diagnosis of the case. He believed that the old doctor's death was due to the development of a gummatous tumor which resulted in hemorrhage at the base of the brain, the hemorrhage no doubt giving rise to the sudden and profound coma which preceded the end. The patient had objections to taking mercury, and instead had taken large quantities of McDade's remedy, together with iodide of potash.

Dr. Nicolson presented a specimen of dermoid cyst of the ovary. He reported the case as one of interest, especially on account of difficulties presented in the matter of diagnosis, it having been mistaken for a cyst of the pancreas. The history of the case as presented is briefly as follows: A little girl twelve years of age, owing to a fall eighteen months ago, was confined to her bed for a week or ten days, suffering pain in the umbilical region. After that time a sort of indurated swelling was felt between the umbilicus and epigastrium, which gradually increased until the whole abdomen became enlarged, presenting a distended appearance, and was regarded by the attending physician, a gentleman from South Carolina, as an echinococcus cyst of the liver. The health of the child remained fair, although she was naturally frail. The last two months the tumor seemed to develop quite rapidly, causing the abdominal walls to become tense so that the tumor could not be outlined. Dr. Gaston then saw the case with him, and upon examination they were unable to decide as to the character of the cyst. They came to the conclusion that it must be either an echinococcus cyst of the liver or a cyst involving the pancreas or kidney. At a subsequent examination a hypodermic needle was introduced and a small quantity of the fluid drawn off by means of a hyperdermic syringe. The movements of the diaphragm, occasioned by the crying of the child, caused the needle to move rapidly up and down which showed that the cyst wall was unattached anteriorly. Microscopical examination of the fluid excluded echinococcus cyst, and the fact that the fluid emulsified fat, contained cholesterin plates, blood corpuscles and other cells, about which he unfortunately misunderstood Dr. Wile, who made the examination, together with the history of the case, led him to give up the idea of an ovarian cyst. Dr. Gaston suggested the cyst as having a pancreatic origin, several interesting cases of the kind having been reported recently, one very noted case, operated on by Dr. Bozeman, for ovarian cyst, which turned out to be cyst of the pancreas. Having decided that it was a cyst of the pancreas, an operation was deemed advisable. An incision was made four inches in length in the median line between the umbilicus and the ensiform cartilage, under strict antiseptic precautions. The cyst was opened and eighteen pounds of fluid removed. It was impossible to draw the cyst up more than a few inches. A sound introduced into the cavity of the cyst revealed much solid matter having a bony character and numerous irregular cavities in different parts. Afterwards the hand was introduced and the cyst was found to be practically immovable. The cyst wall was attached tc the walls of the abdomen by means of catgut ligatures and the external wound was closed with the exception of a small space through which a drainage tube was inserted. The patient rallied and there was little subsequent rise of temperature, 102 degrees being the highest point reached during the first seven days. The upper part of the wound seemed to heal by first intention. On the seventh day the tube was with some difficulty removed, and the day following, the temperature arose to 104 degrees. Two days later the patient died from exhaustion, and the *post-mortem* examination revealed an ovarian cyst of a dermoid character and of unusual size. In removing the cyst it was necessary to break up adhesion between the cyst wall and the intestines, bladder and uterus. The specimen was then presented to the Society, and on examination showed numerous larger and smaller cavities containing hair, teeth, irregular masses of bone and sebaceous material.

Dr. Gaston thought that the history of the case was very misleading, and, together with the points already mentioned by Dr. Nicolson, led to an exclusion of ovarian cyst. The idea of a pancreatic cysts was entertained on account of the position and apparent direction of the development of the cyst, together with a recent investigation on the subject of pancreatic cysts, especially in the light of a paper on this subject published lately by Dr. Senn, of Chicago. He then read extracts from this paper.

Dr. Bizzell stated that he was present at the operation, and from a superficial examination of the patient, and the appearance of the fluid, he at the time expressed his doubts as to the diagnosis, believing the cyst to be of ovarian origin. He based his conclusion on the age of the patient, the extreme rarity of pancreatic cysts, and he felt confirmed in his belief from a subsequent microscopic examination of the fluid, in which he found the ovarian corpuscle of Drysdale. He then gave an able resumé of the development of dermoid cysts of the ovary, mentioning also the blastodermic origin of the different structures found in the cyst.

Dr. Wilson asked Dr. Bizzell whether, in his opinion, there would have been a tolerably good chance of recovery if ovariotomy had been performed in this case.

Dr. Bizzell answered that if we had operators like Luecke and Lawson Tait, who performed these operations as they came, be the cases favorable or unfavorable, and with such a remarkably low rate of mortality, there might have been a different termination had the cyst been extracted in this case.

Dr. Hardon asked whether all the means of diagnosis had been exhausted before coming to the conclusion as to the nature of the cyst; whether examination had been made by the rectum.

Dr. Nicolson said that he thought that the age of the patient precluded such procedure.

Dr. Hardon stated that he had made this kind of examination in a child three years of age. Such an examination often revealed pelvic attachments in cases of ovarian cyst. He also asked if the fluid was examined for para and meta albumen, as these chemical tests were regarded as pathognomonic of ovarian cyst.

Dr. Nicolson answered in the negative.

Dr. Hardon thought that in such cases all the means at hand should be exhausted before arriving at the diagnosis, but that it was rather an ungracious matter, however, to criticise the diagnosis of a colleague after the termination of a case.

Dr. Todd coincided with the views of Dr. Hardon as to the necessity of exercising charity toward each other in mistakes of diagnosis. He thought that abdominal tumors were very difficult, as a rule, to diagnose, and that the microscopic examination of the fluids had thrown very little light on the subject. He spoke of the necessity and value of *post-mortem* examinations as a means of discovering the exact nature of such cases.

Dr. Olmstead also spoke of the difficulty of correctly diagnosing abdominal tumors, and quoted the remark of Spencer Wells, who, after performing five hundred operations, said that no man knew what he was going to find when he opened the abdomen.

Dr. Nicolson said, in conclusion, that he felt very little compunction as to the results of this case, as surgeons of great note have been known to make mistakes of this kind. He had been misled by the history of the case, and relied too much on the statement of the other physicians that the tumor had developed from above downward. He thought Dr. Bizzell a little too positive in his assertions, and that the remarks made by that gentleman as to the diagnosis hardly coincided with the fact, that before the *post-mortem*, the doctor had exhibited a drawing of colloid cells which were claimed to have been discovered in the little particles floating in the fluid, and which had led him to suspect the tumor as being of a colloid, cancerous character, shaking his faith at that time in its ovarian nature. He was not aware that the gentleman had any positive diagnosis at the time. The case was presented in the hope that it would be a useful lesson to the Society, and as an illustration of the difficulty which abdominal tumors, as a rule, present in the matter of diagnosis.

Dr. Todd presented the following formula for a cough medicine in cases of phthisis, which had recently been reported in some medical journal, and which, after trial in twenty cases, had been found to give satisfaction. Formula: Fluid extract of yerba santa; fluid extract grindelia robusta, simple syrup; syrup of tolu, each one ounce. One teaspoonful *pro re nata*.

On motion, the Society adjourned.

