# A Correction

**Published:** 1886-03

**Authors:** 


A CORRECTION.—In the February issue of THE JOURNAL, page 749, second line from top, for eczema *fissures* read eczema *fissum.*

